# Microorganisms in chemotherapy for pancreatic cancer: An overview of current research and future directions

**DOI:** 10.7150/ijbs.59117

**Published:** 2021-06-26

**Authors:** Si-Yuan Lu, Jie Hua, Jin Xu, Miao-Yan Wei, Chen Liang, Qing-Cai Meng, Jiang Liu, Bo Zhang, Wei Wang, Xian-Jun Yu, Si Shi

**Affiliations:** 1 Department of Pancreatic Surgery, Fudan University Shanghai Cancer Center, Shanghai, China.; 2 Department of Oncology, Shanghai Medical College, Fudan University, Shanghai, China.; 3 Shanghai Pancreatic Cancer Institute, Shanghai, China.; 4 Pancreatic Cancer Institute, Fudan University, Shanghai, China.

**Keywords:** Pancreatic cancer, Oncolytic viruses, Bacteria, Mycoplasma, Chemotherapy

## Abstract

Pancreatic cancer is a malignant tumor of the digestive system with a very high mortality rate. While gemcitabine-based chemotherapy is the predominant treatment for terminal pancreatic cancer, its therapeutic effect is not satisfactory. Recently, many studies have found that microorganisms not only play a consequential role in the occurrence and progression of pancreatic cancer but also modulate the effect of chemotherapy to some extent. Moreover, microorganisms may become an important biomarker for predicting pancreatic carcinogenesis and detecting the prognosis of pancreatic cancer. However, the existing experimental literature is not sufficient or convincing. Therefore, further exploration and experiments are imperative to understanding the mechanism underlying the interaction between microorganisms and pancreatic cancer. In this review, we primarily summarize and discuss the influences of oncolytic viruses and bacteria on pancreatic cancer chemotherapy because these are the two types of microorganisms that are most often studied. We focus on some potential methods specific to these two types of microorganisms that can be used to improve the efficacy of chemotherapy in pancreatic cancer therapy.

## Introduction

Pancreatic cancer is one of the most destructive and lethal malignant neoplasms. According to data from the authoritative American Medical Association, the numbers of new male and female clinical cases of pancreatic cancer rank as the tenth and ninth highest, respectively, among all new tumor cases, and more critically, the number of deaths from pancreatic cancer ranks 4^th^ among all deaths from carcinoma. However, more than 80% of pancreatic cancer patients have already lost the opportunity to receive surgery when they are diagnosed. This is a possible explanation for the extremely low five-year survival rate (less than 9%) among pancreatic cancer patients 1. Since the Food and Drug Administration (FDA) approved the use of gemcitabine in 1996, this drug has been widely used to treat breast cancer, lymphoma, ovarian cancer and other tumors and is the cornerstone of pancreatic cancer chemotherapy 2. Unfortunately, patients often develop gemcitabine resistance within a few weeks of chemotherapy. Changes in drug metabolism, reduced apoptosis, and the effects of the tumor stroma are all fundamental causes of drug resistance 2. Therefore, many scientists have begun to develop new methods of pancreatic cancer therapy, including the application of nanotechnology 3, strategies targeting tumor metabolism 4, 5, immunotherapy 6, stem cell therapy, strategies targeting the stroma 7, and strategies targeting signaling pathways 8. Currently, the pancreatic cancer tumor microenvironment is a popular research topic. Many investigators have noted that microorganisms are closely related to the occurrence and progression of pancreatic cancer 9-11. Other studies have shown that microorganisms are involved in chemotherapy resistance or can aid in its effect 12-14. While the underlying mechanisms are still being elucidated, the role of microorganisms in chemotherapy deserves to be explored further. This article primarily addresses the effects of microorganisms on carcinogenesis and chemotherapy in pancreatic cancer. Summarizing the benefits and detriments of these microorganisms may aid in the identification of new directions and different research ideas to improve the survival rates of pancreatic cancer patients.

## Microorganisms and pancreatic cancer tumorigenesis

**Figure 1:**
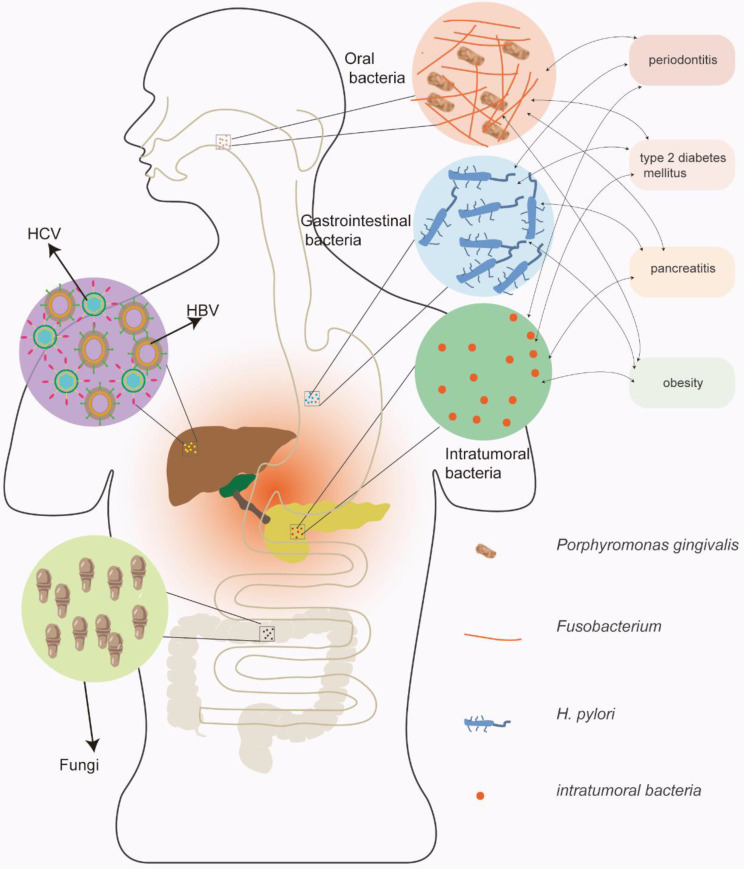
** The relationship between microorganisms and pancreatic cancer.** Viruses, bacteria, and fungi may all contribute to the carcinogenesis of pancreatic cancer. Among the pathogenic pathogens, the viruses primarily include hepatitis B and hepatitis C viruses; the bacteria primarily include oral and gastrointestinal bacteria. In addition, bacteria have complex interactions with risk factors for pancreatic cancer.

Many studies have demonstrated the potential role of microorganisms in pancreatic cancer carcinogenesis. Chronic inflammation primarily induced by microorganisms may be a vital mechanism, and microorganisms can also change the immune microenvironment and regulate the hallmarks of pancreatic cancer 15. The relationship between microorganisms and pancreatic cancer is summarized in Figure 1; viruses, bacteria, and fungi may all contribute to the carcinogenesis of pancreatic cancer.

### Virus and pancreatic cancer tumorigenesis

Many viruses are thought to be related to cancer carcinogenesis, including human papilloma virus (related to cervical cancer), Epstein-Barr virus (related to nasopharyngeal carcinoma) and hepatitis virus (related to liver cancer). Interestingly, hepatitis virus may also be correlated with pancreatic cancer, particularly hepatitis B virus (HBV) and hepatitis C virus (HCV) 16. HBV and HCV, typical hepatotropic viruses, can not only appear in the liver but can also be detected in the pancreas 16. Some researchers detected HBV in the pancreatic acinar and pancreatic juice of HBV patients, and a correlation with pancreatitis was found 17. In addition, studies have shown that people with HBV or HCV have a higher risk of pancreatic cancer than those without hepatitis 18, 19. The potential mechanisms by which HBV and HCV promote pancreatic cancer occurrence may include persistent chronic inflammation and changes in tissue elasticity 17, 20. Some investigators have proposed that the HBx protein expressed by HBV may induce pancreatic cancer carcinogenesis through the PI3K/AKT signaling pathway. However, this induction may only explain a small part of the underlying mechanism, and more investigations are still needed to explore the inner relationships between viruses and pancreatic cancer.

### Bacteria and pancreatic cancer tumorigenesis

Bacteria are critical carcinogens and have been investigated in many tumors, including pancreatic cancer. Oral bacteria, including *Porphyromonas gingivalis (P. gingivalis*) 21 and *Fusobacterium* 22, gastric and intestinal bacteria, including *H. pylori* 23 and some intratumoral bacteria, may play a significant role in the occurrence of pancreatic cancer. These bacteria, which may reflux to the pancreas along the digestive tract, have been detected in pancreatic cancer specimens during many studies. A high level of *P. gingivalis* was correlated with a 2-fold increased risk of pancreatic cancer occurrence 22. Similarly, *H. pylori* was found to be positively related to pancreatic cancer, and Helicobacter DNA was confirmed in tumor tissues but not in normal tissues 24. In addition, some pancreatic cancer specimens were found to have elevated bacterial abundance compared to normal tissues, indicating their effects on pancreatic carcinogenesis 25. Some regulatory mechanisms in inflammation and immune microenvironments were elucidated when researchers explored the correlation between bacteria and pancreatic cancer tumorigenesis. The bacterial pathogens remaining in the oral and gastrointestinal tracts can always lead to local inflammation and induce the production of inflammatory factors, including interleukins, tumor necrosis factor, and some kinases 26, further causing the activation of tumor-related signaling pathways and the development of important tumor hallmarks 10. In addition, some studies have shown that gut bacteria can upregulate TLR receptors in pancreatic cancer and induce immune tolerance 27, playing an important role in regulating the tumor microenvironment.

Some risk factors for pancreatic cancer may also interact with bacteria in the digestive tract, including periodontitis, type 2 diabetes mellitus, pancreatitis and obesity. Periodontitis, which is always caused by *P. gingivalis* and *Fusobacterium*, has been shown to be a critical risk factor for pancreatic cancer 28. While periodontitis pathogens destroy the oral health environment and cause chronic inflammation of the oral cavity, they may also promote* p53* and *K-ras* mutations 29, promoting the occurrence of pancreatic cancer. Intestinal bacteria may also influence the pathophysiological process of type 2 diabetes 30. Low levels of beneficial bacteria may reportedly cause intestinal inflammation and insulin resistance 30. In addition, intestinal bacteria can also affect the metabolism of short-chain fatty acids 31, inhibit bile acid synthesis and disrupt the intestinal barrier 32, promoting the development of type 2 diabetes to some extent. Obesity, which is currently thought to be a risk factor for pancreatic cancer, is also regulated by intestinal bacteria. Some studies have indicated that the number of *Bacteroidetes* in obese mice is decreased and the level of *Firmicutes* is increased 33. Interestingly, a similar phenomenon was observed in human subjects 34. The diversity of the bacteria in obese patients is lower than that of normal-weight people 35, suggesting that bacteria may be involved in the pathogenesis of obesity. Another risk factor affected by bacteria includes pancreatitis. Although there may be no bacterial infection during the early stage of acute pancreatitis, bacterial infection can induce persistent inflammation and even pancreatic necrosis in the late stage of pancreatitis 36. In addition, the microbiota composition of chronic pancreatitis patients changes significantly compared to that of people without pancreatitis 37. Downregulated *Actinobacteria* abundance and upregulated *Escherichia-Shigella* abundance are observed, and the diversity and coordination of gut microbiota are decreased 37.

### Fungi and pancreatic cancer tumorigenesis

Fungi, a component of the gut microbiota, have not received much attention from researchers. Although fungi are much lower than bacteria in terms of flora number and abundance, they may play an equally important role as bacteria in pancreatic cancer oncogenesis 38. Aykut et al found that the number of fungi in pancreatic cancer tissues and mouse models was 3000 times higher than that in normal tissues, with a remarkable enrichment effect on *Malassezia spp.* 39. Interestingly, scientists also found *Malassezia spp*. in the oral cavity, which also confirmed the important role of oral pathogens in the occurrence of pancreatic cancer 40. Further studies suggested that the mechanisms by which fungi promote pancreatic cancer progression may lie in the MBL-C3 pathways, as verified in *Malassezia spp.* 39. In addition, the interaction between bacteria and fungi is also worth exploring. Some researchers have shown that fungi may participate in the bacterial immune response by activating C3 and that bacteria may also regulate MBL-C3 pathways through the immune response 41. The mutual regulation of bacteria and fungi will make it more complicated to identify the carcinogenic mechanisms.

## Viruses and pancreatic cancer chemotherapy

Viruses are pathogens that cause various detrimental effects on physical and mental health. Of the nearly one million vertebrate viruses, approximately 320,000 can infect mammalian cells 42. Research on the mutual effect of common viruses that influence pancreatic cancer chemotherapy has not yet been reported. However, oncolytic viruses, either as genetically engineered or naturally occurring viruses, have become subjects of note for their interaction with chemotherapy. Bischoff et al reported the first oncolytic virus, dl1520, which can replicate selectively in p53-deficient human tumor cells 43. This discovery caused a great upsurge in research on oncolytic viruses in the scientific community, which led to the exploration of a series of oncolytic viruses for targeting various tumors. Furthermore, oncolytic virus therapy has recently been recognized as a promising new therapeutic approach to cancer treatment 44-56. Surprisingly, many oncolytic viruses that act against pancreatic cancer are being researched, and when used in combination with gemcitabine, they can significantly assist in the killing of pancreatic cancer cells and dramatically improve the effect of chemotherapy. This part of the review will describe the latest results of combining oncolytic viruses with chemotherapy (as represented by gemcitabine) according to the category of virus in use, with a focus on the underlying mechanisms to explore better combination therapies.

### Adenoviruses and pancreatic cancer chemotherapy

Adenoviruses are small, non-enveloped double-stranded icosahedral DNA viruses, and there are >57 serotypes of adenoviruses that are classified into subtypes A-G based on their respective agglutination properties 57, 58. Intriguingly, the adenovirus can function as both a pathogen and a therapeutic tool 59-61. Every oncolytic adenovirus is essentially produced by deleting viral genes, including *E1B55K, E1B19K,* and* E1ACR2*, and the majority of oncolytic adenoviruses are based on adenovirus serotype 5 (Ad5), which has been shown to be safe in cancer patients and to eliminate cancer cells with limited toxicity to healthy cells specifically 62. Studies have shown that most oncolytic adenoviruses can produce a sensitization effect when combined with gemcitabine and can enhance the killing effect on pancreatic cancer cells 63. The first studied oncolytic adenovirus was onyx-015, which carried a deletion of the *E1B55K* gene 43. The *E1B55K* gene is found within the E1B region of the viral genome. It can combine with the *p53* gene and inactivate it. Due to its deletion, the oncolytic adenovirus can replicate in tumor cells but cannot replicate in normal cells. This property guarantees the safety of the oncolytic adenovirus 43. Experiments using cell lines and tumor-bearing nude mice showed that onyx-015 efficiently lyses pancreatic cancer cells deficient in* p53* expression, and some scientists found that infected pancreatic cancer cells can express viral antigens on the surface, resulting in a host immune response to reinforce antitumor immunity 43. However, Hecht and Mulvihill et al. evaluated the effects of ONYX-015 combined with gemcitabine on pancreatic cancer cells, and the results were not satisfactory 64, 65. This poor result may have occurred because the deletion of* E1B55K* weakened the ability of the virus to replicate and spread 66, 67. As a result, scientists began to explore new excision sites, and on this basis, they developed new oncolytic adenovirus mutants accompanied by different gene deletions, for example, AdΔE1B19K (*E1B19K* deleted), AdΔΔ (*E1ACR2* and* E1B19K* deleted), AdΔCR2 (*E1ACR2* deleted), dl312 (*E1A* and *E3B* deleted) and dl922-947 (*E1ACR2* and *E3B* deleted) 63, 68, 69. These newly developed oncolytic viruses have shown exciting effectiveness. Oncolytic adenoviruses with *E1B19K* gene deletion can achieve valid targeting at a dose lower than the clinically toxic dose when used in combination with chemotherapy. The enhanced antitumor effect is due to the enhanced drug-induced apoptosis induced by synergism between the virus and gemcitabine. Gemcitabine induces tumor cell death through classical apoptosis, and adenovirus activates tumor cell death through nonapoptotic pathways 68. Interestingly, scientists have found that oncolytic adenovirus mutants such as dl922-947, from which *E1ACR2* was deleted*,* are more efficient than ONYX-015, but they are more toxic 69-73. AdΔΔ is an oncolytic mutant with both *E1ACR2* and *E1B19K* deleted. Compared with wild-type adenovirus, ONYX-015 and dl922-947, AdΔΔ retains the ability to sensitize cancer cells in combination with cytotoxic drugs, and when combined with gemcitabine, it not only induces more apoptosis to effectively kill tumor cells but also has lower toxicity than when it is used as a monotherapy 63, 68, 74. However, to treat pancreatic cancer more briefly and effectively in combination with chemotherapy through systemic administration, adenovirus mutants must be further modified for the following reasons: (1) coxsackie virus receptors, natural virus receptors and adenovirus receptors, which are expressed at a high level in human erythrocytes, can bind to the fiber knob of the virus^75^; and (2) the high-affinity binding to numerous blood factors and Kupffer cells in the liver can quickly clear the virus 62, 76. To improve the tumor-killing and chemotherapy synergistic effects, some researchers modified AdΔΔ to achieve increased targeting. Compared with normal tissue cells, most cancer cells can express integrin avβ6, which is a useful target. Therefore, they added the A20FMDV2 peptide to target the Arg-Gly-Asp (RGD) domain of integrin avβ6 selectively and deleted *E3gp19k*, resulting in a new mutant, Ad-5-3Δ-A20T, which was more lethal than AdΔΔ against pancreatic cancer when combined with gemcitabine. Its primary mechanisms lie in its increased recruitment of immune cells to infected tumor cells and its enhancement of gemcitabine-dependent apoptosis 77. To date, the most promising mutants for pancreatic cancer treatment are LOAd703 and VCN-01. LOAd703 has a deleted *EIACR2* region to improve tumor selectivity and has an *E2F* binding site inserted upstream of *E1A* to control virus replication; in addition, its most critical feature is an insertion of the trimerized, membrane-bound human CD40 ligand (TMZ-CD40L) and the full-length human 4-1BB ligand (4-1BBL) 78-80. CD40L activates apoptosis in cancer cells and increases myeloid and T cell infiltration to increase the antitumor effect. 4-1BBL can increase the infiltration of lymphocytes into tumors and enhance the CD40/CD40L synergistic antitumor immune response 78, 81, 82. VCN-01 also has a deleted E1ACR2 region and an inserted E2F-binding site 83, 84. The replacement of the KKTK binding site in VCN-01 with an RGD domain prevents the effects of adenovirus on hepatocytes. The addition of PH20 hyaluronidase facilitates the spread of adenovirus in the interstitium of connective tissue and the extracellular matrix and promotes the infiltration of immune cells 85-89. Currently, relevant clinical trials of VCN-01 and LOAd703 are ongoing, and researchers have added gemcitabine to their list of intervention measures.

### Herpes simplex virus type 1 and pancreatic cancer chemotherapy

Herpes simplex viruses (HSVs) can be divided into type 1 and type 2. HSV-1 is an enveloped double-stranded DNA virus that has the following merits and can be used as an oncolytic virus 90-92: (1) It has a wide range of infection targets and can infect almost all types of human cells and various tumor cell types 92. (2) Almost all HSV-1 gene sequences have been discovered, and they are long and suitable for the insertion of foreign genes 93, 94. (3) HSV-1 infectivity is higher than that of adenovirus and adeno-associated virus 95. (4) Oncolytic viruses based on HSV-1 are very safe. Although they are associated with some adverse events, there are sensitive anti-HSV drugs, such as ganciclovir and acyclovir. Because HSV-1 has good oncolytic activity, many scientists have used it for treating pancreatic cancer, and in combination therapy with chemotherapy, it has achieved beneficial synergistic effects. G207 is a second-generation mutant of HSV-1 with a deletion of the *γ_1_34.5* gene and an insertion of the *E. coli. lacZ* gene in the infected cell polypeptide 6 (ICP-6)-coding region 96, 97. The insertion of the *E. coli lacZ* gene enables G207 to replicate more efficiently than HSV-1 in nondividing cells 96, 97. To explore the therapeutic effect of G207 in pancreatic cancer, Lee et al. conducted experiments on three pancreatic cancer cell lines, ASPC-1, MIA Paca-2 and BxPC-3, *in vitro*. Their study confirmed the infection, replication and killing abilities of G207 and was the first to demonstrate the great potential of G207 for treating pancreatic cancer 98. In addition, scientists have begun to explore chemotherapy combination strategies. Martuza and Samuel et al. found that when HSV-1-based viruses such as G207 and NV1020 are combined with traditional chemotherapy, their killing effect on pancreatic cancer cells is greatly enhanced, providing a promising clinical treatment strategy^99^. However, some of the oncolytic HSVs mentioned above have some limitations in terms of their tumor-killing ability and replication efficiency. With the development of science and technology, many new mutants have emerged. Myb34.5 is a typical mutant used in pancreatic cancer studies in which the expression of ICP6 is defective and the expression of *γ_1_34.5* is driven by the B-myb promoter 100. In an experimental model of pancreatic cancer, mice that received an intratumoral injection of Myb34.5 had a good prognosis. Their tumors were necrotic and showed bleeding, and their cancer cells died due to apoptosis 101. The synergy between Myb34.5 and chemotherapy drugs is intriguing. The combined use of low-dose virus and gemcitabine can significantly kill MIA Paca-2 pancreatic cancer cells *in vitro* and is much more effective than the use of gemcitabine alone 101. Interestingly, the killing effect of Myb34.5 on tumors depends on virus replication rather than the host immune response, according to some studies 101. HF10 is an oncolytic virus derived from HSV-1 that is currently a popular topic of research. It has a strong tumor-killing ability but does not damage normal tissues 102. Previous clinical studies have shown that HF10 increases the number of CD4+, CD8+ and natural killer cells in tumors, which may slow tumor growth and prolong survival rates 95, 102, 103. Recently, in a clinical trial on 12 patients with unresectable pancreatic cancer, HF10 combined with gemcitabine and erlotinib showed a higher antitumor effect than HF10 alone. Among the 12 patients, 3 had partial remission, 4 had stable disease, and the total effective rate was 78% 104. Although the mechanism by which HF10 interacts with chemotherapy is still unclear, the role of HF10 in combination therapy for pancreatic cancer deserves further exploration.

### Vaccinia viruses and pancreatic cancer chemotherapy

Vaccinia viruses have been widely known since their application in preventing smallpox. Recently, due to their tropism for cancer cells, easy genetic modification and other biological characteristics, vaccinia viruses have become a popular choice for use as oncolytic viruses 105, 106. They have shown good antitumor activity and the ability to replicate selectively in tumors in* in vivo* and* in vitro* models of pancreatic cancer. More specifically, many scientists have discovered their beneficial synergistic effects when combined with gemcitabine for treating pancreatic cancer. ING4 is a protein that can inhibit angiogenesis and increase the sensitivity of cancer cells to chemotherapy; undoubtedly, it is a highly effective protein that can be used to treat pancreatic cancer 107, 108. Wu et al. constructed a new oncolytic virus, VV-ING4, which contains the gene encoding ING4, and this oncolytic virus had a stronger cytotoxic effect than the original virus and could induce pancreatic cancer cell apoptosis and G2/M phase arrest. When VV-ING4 and gemcitabine were used synergistically in *in vitro* experiments, they significantly inhibited the replication of SW1990 and PANC-1 pancreatic cancer cells. This result may have occurred because VV-ING4 improves chemotherapy sensitivity and promotes the penetration of gemcitabine 109. Because sensitizing proteins are a hot research topic, research into the antigenicity of tumors is another direction for scientists to identify novel therapeutic strategies. Survivin, a tumor-associated antigen, is overexpressed in most pancreatic cancer cells and lacks expression in most differentiated and mature cells. Increased survivin expression is related to increased activity of cancer cells, resistance to cancer treatments and tumor progression. A modified vaccinia virus Ankara (MVA-survivin) expressing survivin was constructed by Ishizaki et al., who also explored its effect on pancreatic cancer models in combination with gemcitabine 110. The experimental results showed that when MVA-survivin and gemcitabine were used in combination to treat Pan02 tumors, the tumors regressed significantly, and the survival time was prolonged. This finding may be related to the enhancement of related antitumor immunity 110.

Interestingly, in research on the combined application of GLV-1h68 (an oncolytic vaccinia virus) and chemotherapy drugs in pancreatic cancer, different scientists have obtained slightly different results. Yu et al. found that the tumor-killing effect was significantly enhanced when GLV-1h68 was combined with gemcitabine to treat PANC-1 tumors 111. However, in another study on GLV-1h68 combined with nab-paclitaxel plus gemcitabine to treat pancreatic cancer, scientists found that the cytotoxicity of GLV-1h68 combined with gemcitabine treatment was not increased compared with that of the single treatment. In addition, in GLV-1h68 combined with nab-paclitaxel plus gemcitabine triple therapy, the cytotoxicity to BxPC-3 and MIA Paca-2 tumor cells was significantly reinforced 112. Therefore, we hypothesize that vaccinia viruses do have beneficial synergistic effects in combination with chemotherapy for treating pancreatic cancer, and determining the most effective combination strategy is worthy of further exploration.

### Other oncolytic viruses and pancreatic cancer chemotherapy

In addition to the commonly used oncolytic viruses that have been introduced above, there are several oncolytic viruses that have shown to have increasing potential for treating pancreatic cancer, including Newcastle disease virus, measles virus, myxoma virus and vesicular stomatitis virus.

Newcastle disease virus belongs to the Paramyxoviridae family and is a naturally occurring negative-sense single-stranded RNA virus that has been studied for many years. Its genome contains 6 genes encoding eight proteins that can efficiently and selectively kill many types of human tumor cells 113. Early clinical trials have found that several naturally occurring Newcastle disease viruses have the ability to kill pancreatic cancer tumors, but the effect is minimal. Therefore, some scientists continue to use genetic engineering techniques to transform Newcastle disease viruses to achieve better oncolytic effects 114, 115. Buijs et al. 116 evaluated the response of 11 different human pancreatic cancer cell lines to Newcastle disease virus infection and interferon treatment and found that improving the anti-interferon properties of Newcastle disease virus may enhance its oncolytic effect. At present, the combination of Newcastle disease virus and chemotherapy for treating pancreatic cancer has not been explored, and it may achieve surprising results 117. Interestingly, a combination of measles virus and chemotherapy has been reported. Some scientists found that the combined application of a small amount of measles virus and a subtherapeutic concentration of gemcitabine could reduce the mass of pancreatic cancer cells by more than 50%, and measles virus and gemcitabine were shown to have synergistic effects, greatly improving the oncolytic activity 118. To explore the synergy of measles virus and gemcitabine, Bossow et al. designed a completely redirected measles virus, namely, MV-PNP-anti-PSCA. It can target prostate stem cell antigen (PSCA; which can be expressed in pancreatic cancer) and carries the gene encoding the prodrug-converting enzyme PNP. This new oncolytic measles virus can specifically infect pancreatic cancer cells and shows high oncolytic activity in cell lines that express PSCA. Importantly, in pancreatic cancer cells resistant to gemcitabine, cross-resistance to this mutant has not been detected. This finding is undoubtedly good news for pancreatic cancer patients who are resistant to chemotherapy 119. Myxoma virus is a member of the poxvirus family. Although it is considered nearly harmless to normal human cells, it has a good killing effect on pancreatic cancer cells. Resistance to gemcitabine is related to increased levels of activated Akt, and the upregulation of phosphorylated Akt enhances productive infection by myxoma virus, suggesting that myxoma virus may be a potential alternative therapy for pancreatic cancer, especially for those resistant to gemcitabine 120-122. *In vivo* and *in vitro* studies have shown that the use of myxoma virus and gemcitabine sequential therapy on the Hs766T and Pan02 pancreatic cancer cell lines improves the overall survival rate of mice. In Hs766T tumor cells, treating with gemcitabine followed by oncolytic virus is more effective than either agent as a monotherapy. In the Pan02 cell line, treating with oncolytic virus followed by gemcitabine had better results than either agent as a monotherapy 123. Vesicular stomatitis virus is also very popular for treating pancreatic cancer. Vesicular stomatitis virus is a non-segmented, negative-strand RNA virus and a promising oncolyte that can kill pancreatic cancer cells by inducing apoptosis 124. VSV-ΔM51-GFP is a new type of vesicular stomatitis virus that can be used as a chemotherapy sensitizer. VSV-ΔM51-GFP has a methionine deletion at amino acid 51 of the matrix protein and a green fluorescent protein (GFP) open reading frame (ORF) inserted at position 5 of the viral genome, which can activate both the endogenous and exogenous apoptotic pathways to induce pancreatic tumor cell apoptosis; compared to other viruses, it is a more effective oncolytic agent 125. When VSV-ΔM51-GFP was combined with gemcitabine for pancreatic cancer treatment, a significant improvement in the therapeutic effect was observed. This finding suggests that vesicular stomatitis virus has a promising future in combination with gemcitabine 126.

### Underlying mechanisms of synergism between oncolytic viruses and chemotherapy

**Figure 2:**
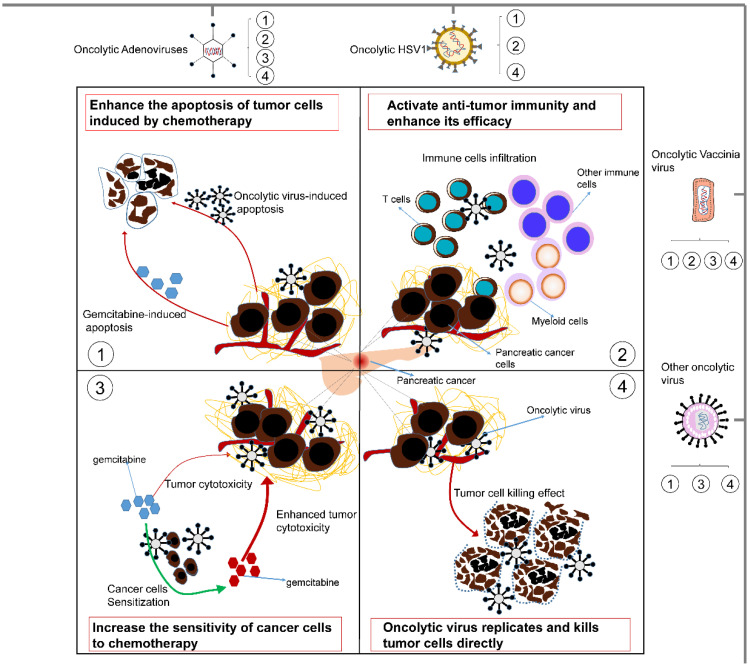
** Some underlying mechanisms by which oncolytic viruses influence the treatment effect of chemotherapy.** 1) Oncolytic viruses can enhance the apoptosis of tumor cells induced by chemotherapy. Moreover, they can also induce tumor cell apoptosis by themselves. 2) Oncolytic viruses can activate antitumor immunity and enhance its efficacy. They can cause the infiltration of T cells, myeloid cells and other immune cells in the tumor to enhance the antitumor activity of chemotherapy. 3) Oncolytic viruses can increase the sensitivity of tumor cells to chemotherapy, thereby making tumor cells easier to kill. 4) Oncolytic viruses can replicate and multiply only in tumor cells, thereby directly killing tumor cells and complementing the killing effect of chemotherapy. Different oncolytic viruses contain different synergistic mechanisms, but they all improve the tumoricidal effect of chemotherapy to varying degrees.

Although substantial evidence indicates that oncolytic viruses can have a good synergistic effect with chemotherapy, the specific molecular basis has not yet been summarized. In this section, we prioritize concrete mechanisms that may involve direct oncolysis, increasing the sensitivity of cancer cells to chemotherapy and activating antitumor immunity and the apoptosis of tumor cells. These important mechanisms are summarized in Fig. 2.

#### Direct tumor-killing effect

The oncolytic effect of oncolytic viruses is their foremost property. Oncolytic viruses can selectively invade pancreatic cancer tumor cells, leading to their cracking and death, which in turn leads to increasing positive feedback for lysis. Normal cells have complete antiviral immunity, and oncolytic viruses are quickly cleared when they try to invade normal cells. However, the antiviral capabilities of tumor cells are defective, and they may have defects in the PI3K/AKT signaling pathway or tumor suppressor genes such as *P53* and *RB* 127, which often makes tumor cells more sensitive to oncolytic viruses.

#### Enhance tumor sensitivity to chemotherapy

The biggest flaw of chemotherapy is its easily induced tolerance, and oncolytic viruses solve this drawback to a certain extent. Deleting the *E1B19K* gene can make pancreatic cancer cells sensitive to chemotherapy-induced death 68. Oncolytic viruses primarily act on cell cycle regulation, increase the DNA damage induced by chemotherapy and exert sensitization effects. They can attenuate the activation of Chk1 and the DNA repair factor Mre11 128. Another important mechanism is to prevent the drug-induced accumulation of Claspin, which is a required protein for Chk1 activation. These processes lead to a virus-mediated reduction in the DNA damage response (DDR) and eventually to sensitization 128. Another sensitization mechanism is related to the extracellular matrix (ECM). The ECM is often distributed on the surface of pancreatic cancers, and its primary components are collagen, fibronectin and elastin 129, which greatly block the effective arrival of chemotherapy. Chemotherapy-mediated drug resistance is also related to ECM-mediated signal transduction, and oncolytic viruses can selectively eliminate abnormal ECM, which can offset ECM-mediated drug resistance and indirectly increase tumor cell sensitivity to chemotherapy 123.

#### Antitumor immunity boosted by the oncolytic virus

Over the past few years, the immune response has been considered to be an obstacle to oncolytic virotherapy. However, we are now aware of the great importance of the immune system in oncolytic virotherapy despite its clearing of oncolytic viruses. Typically, pancreatic cancer generates an immunosuppressive microenvironment that includes immune suppressive cytokines such as interleukin-10 (IL-10) and transforming growth factor β1 (TGFβ1) and immunosuppressive cells, including regulatory T cells (Tregs) and myeloid-derived suppressor cells (MDSCs), leading to the inhibition of the immune response 130. The emergence of oncolytic viruses changes this situation. Oncolytic virus infection can induce direct cell lysis 131 and immunogenic cell death (ICD), which includes pyroptosis 132, 133, necroptosis 134, 135 and autophagic cell death 132, 133. These processes can release cellular damage-associated molecular patterns (DAMPs, for example, heat shock proteins, high mobility group box 1 (HMGB1) protein, calreticulin, and IFN-1), pathogen-associated molecular patterns (PAMPs) and tumor-associated antigens (TAAs) 48. These substances can promote the maturation of antigen-presenting cells (APCs), such as dendritic cells (DCs), and activate the immune response of CD4+ T cells and CD8+ T cells. Activated T cells can produce cytotoxic effects, thereby mediating effective antitumor immune responses 136, 137. In addition, the released cytokines, such as IL-6, IL-8, and IFN-1, can directly activate NK cells and have a direct killing effect on cancer cells 138, 139. Surprisingly, oncolytic viruses can also inhibit the immune escape of pancreatic cancer cells, which is one of the most important hallmarks of cancer. As mentioned above, the tumor immune microenvironment (TME) is a prominent cause of immune escape. Oncolytic viruses can change the cytokines in the TME, such as IL-10 and TGF-β, and the types of recruited immune cells, such as Tregs and myeloid-derived suppressor cells (MDSCs) 140-142, which results in the hyperresponsiveness of tumor cells to the immune system.

#### Induced apoptosis by oncolytic virus

Chemotherapy drugs such as gemcitabine can induce apoptosis in pancreatic cancer tumor cells by damaging DNA and terminating chain synthesis 143. Interestingly, oncolytic viruses can enhance the apoptotic ability of gemcitabine through various additional apoptosis-inducing pathways. In cell cycle regulation, the *P53* and *RB* pathways are two classic pathways that ensure the normal progression of the cell cycle. The occurrence of *P53* mutations in pancreatic cancer cells is the key factor in their unlimited replication capacity. Oncolytic viruses often act on the *P53* pathway to promote the upregulation of Bax and the downregulation of Bcl2 and further activate caspase8/9/3 to cause apoptosis 109, 144. During this process, both the mitochondrial-dependent apoptosis pathway and the death receptor-dependent signaling pathway play a significant role. In addition, there are some other ways of inducing apoptosis that also play an important role. For example, oncolytic viruses can negatively regulate the signal transduction of the NF-κB pathway and other tumor suppressor factors to promote apoptosis 145, 146. Some oncolytic viruses, such as H-1PV, can even establish an apoptotic pathway independent of caspase3 by activating lysosomal proteases and cytosolic relocation 147.

## Bacteria and pancreatic cancer chemotherapy

**Figure 3:**
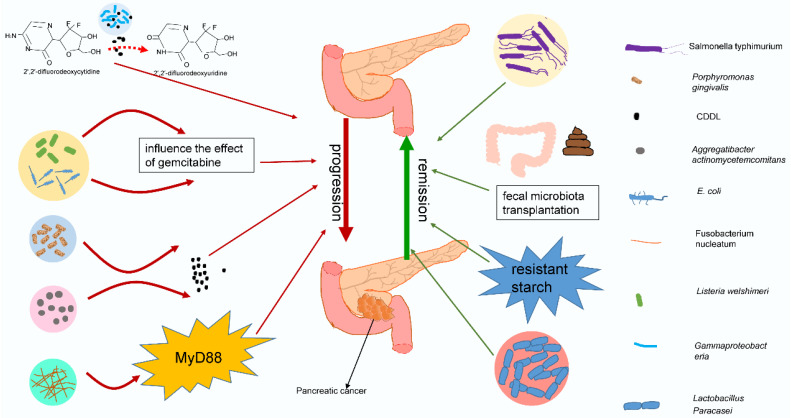
** Different bacteria have different effects on chemotherapy for pancreatic cancer.** Gammaproteobacteria, E. coli, Listeria welshimeri, Porphyromonas gingivalis, Aggregatibacter actinomycetemcomitans, and Fusobacterium nucleatum may have negative effects on pancreatic cancer treatment. They act directly on chemotherapy or indirectly change chemotherapy drugs by secreting enzymes or through signaling pathways, deteriorating the treatment effect of pancreatic cancer. However, Salmonella typhimurium and Lactobacillus paracasei may have beneficial effects on chemotherapy, and fecal microbiota transplantation or resistant starch may improve the treatment efficacy against pancreatic cancer by adjusting the intestinal flora.

**Figure 4:**
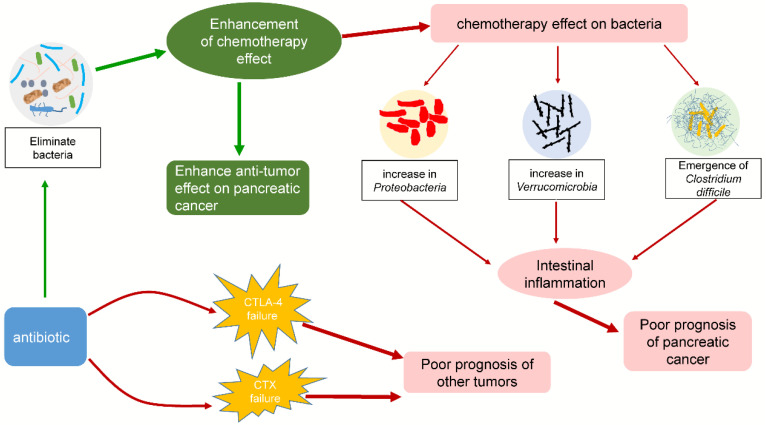
** Some interactions among chemotherapy, bacteria and antibiotics.** Chemotherapy can affect the composition of the intestinal flora and increase the ratio of *Proteobacteria*, *Verrucomicrobia*, and *Clostridium difficile*. These bacteria can cause intestinal inflammation, leading to poor prognosis in pancreatic cancer patients. The addition of antibiotics enhanced the efficacy of chemotherapy by eliminating bacteria, but it also exacerbated the changes in the intestinal flora. Interestingly, while other tumors were being treated with CTLA-4 or CTX in combination with antibiotics, similar consequences were observed.

Recently, many studies have shed light on the potential link between the microbiota and pancreatic cancer 12, 148-150. As an important component of the microbiota, bacteria are inextricably related to the response to chemotherapy (typically gemcitabine) by pancreatic cancer. Some studies have proposed that bacteria in tumors can modulate the chemotherapeutic effect on pancreatic cancer, leading to negative or beneficial effects. Chemotherapy can in turn act on the tumor or intestinal flora, causing various problems. Interestingly, antibiotics also play an important role in chemotherapy for pancreatic cancer by affecting intestinal bacteria. The effects of bacteria on chemotherapy in pancreatic cancer are summarized in Fig. 3. The interactions among chemotherapy, bacteria and antibiotics are summarized in Fig. 4. These findings may provide a basis for using tumor-related bacteria to develop new strategies for treating pancreatic cancer.

### The negative effect of bacteria on pancreatic cancer chemotherapy

Previous studies on the relationship between bacteria and pancreatic cancer have focused on the mechanism by which bacteria give rise to the occurrence and progression of pancreatic cancer 15. Currently, the focus of many studies has begun to shift to the influence of bacteria on chemotherapy in pancreatic cancer. Some evidence shows that the presence of bacteria in the tumor microenvironment will modulate the effectiveness of cancer treatment. Bacteria can metabolize chemotherapy drugs, change their chemical structure, and affect their activity and local concentration 151, 152. Relatively recently, Geller et al. discovered that bacteria can disrupt the metabolism of gemcitabine by pancreatic cancer 150. These researchers examined tumor specimens from 113 pancreatic cancer patients and found bacteria in 76% of tumor specimens, primarily *Gammaproteobacteria*, which can express the long form of CDD (cytidine deaminase) and can metabolize the active form of gemcitabine (2′,2′-difluorodeoxycytidine) into the inactive form, 2′,2′-difluorodeoxyuridine. Interestingly, scientists have observed a similar phenomenon in mycoplasma, which can encode the CDD to inhibit the antitumor activity of gemcitabine in mycoplasma-infected mouse tumor cells, and this inhibition can be relieved by tetrahydrouridine (a cytidine deaminase inhibitor) or antibiotics such as tetracycline 153, suggesting that these bacteria may be related to chemotherapy resistance in pancreatic cancer. An animal model designed to detect the influences of *Escherichia coli (E. coli)* and* Listeria welshimeri* on the efficacy of commonly used chemotherapeutics can be used to support this conjecture partially. The tumor volumes of mice treated with gemcitabine plus bacteria were significantly larger than those of mice treated with gemcitabine alone, indicating that the presence of bacteria had an adverse effect on gemcitabine chemotherapy 151. Surprisingly, some scientists have found that bacteria from other tissues may also be related to chemotherapy resistance. Elevated levels of *Porphyromonas gingivalis* and *Aggregatibacter actinomycetemcomitans* have been detected in patients receiving pancreatic cancer treatment. More specifically, these two bacteria can also express CDD, which indicates that they may be related to chemotherapy resistance in pancreatic cancer 12, 154. Even *Fusobacterium nucleatum (F. nucleatum)*, a bacterium that can cause colorectal cancer to be resistant to oxaliplatin and 5-fluorouracil by affecting the TLR4/MyD88 pathway, has been found to be more abundant in the cancer tissues of patients with pancreatic cancer than in nonpatient controls 155. According to the literature,* F. nucleatum* leads to the loss of miR-18a* and miR-4802 through the TLR4/MYD88 pathway, which leads to the activation of ULK1 and ATG7 and further induces the autophagy pathway, thereby triggering drug resistance in colon cancer. *F. nucleatum* can also cause the immunosuppression of the pancreatic cancer microenvironment through the same MyD88 pathway. Thus, some scientists speculate that this bacterium may also be related to drug resistance in pancreatic cancer based on the similar signaling pathways involved 156, 157.

### The beneficial effects of bacteria on pancreatic cancer chemotherapy

The role of bacteria in pancreatic cancer chemotherapy is not always deleterious, and bacteria can sometimes have a beneficial effect. In a mouse model of pancreatic cancer treated with gemcitabine and bevacizumab, adding *Salmonella typhimurium* improved the therapeutic effect in pancreatic cancer-bearing mice 158. A large cohort study drew a similar conclusion. Researchers found that the diversity of the tumor microbiota in long-term survivors (>5 years) was higher than that in short-term survivors 159. On this basis, many scientists have also begun to investigate strategies that employ bacteria to improve the pancreatic cancer prognosis, and probiotics have become the focus of exploration in new therapies. *Lactobacillus paracasei* is a gram-positive lactic acid bacterium located in the human intestine. Adding *Lactobacillus paracasei* to a mouse model treated with gemcitabine can improve the efficacy of the chemotherapy and increase tolerance 160. Further research showed that it primarily elevated IFN to transfer the Th2 immune phenotype into the Th1 immune phenotype, subsequently enhancing the antitumor ability 161. In addition, some components derived from probiotics also seem to exhibit antitumor activity. The ferrichrome extracted from *Lactobacillus paracasei* can inhibit the proliferation of pancreatic cancer cells and restrain pancreatic cancer cells resistant to 5‑fluorouracil in a mouse model, indicating that the antitumor activity of probiotics may come from the active ingredients in probiotics 162. In addition, probiotics seem to have the ability to reduce adverse postoperative reactions. Postoperative patients taking *Enterococcus faecalis* and *Clostridium butyricum* seem to have fewer infection complications 163. Some methods that can modulate the intestinal flora may also modify the effects of chemotherapy in pancreatic cancer, such as fecal microbiota transplantation, alterations in lifestyle or diet, and the use of prebiotics. Using resistant starch as a prebiotic can facilitate the growth of bacteria involved in butyrate production and delay tumor deterioration in mice bearing pancreatic cancer 164. The application of prebiotics and other modulation methods in treating pancreatic cancer are being explored.

### The effect of chemotherapy on intestinal bacteria and the exploration of antibiotic therapy

The physical condition of cancer patients is generally poor, and the addition of chemotherapy undoubtedly aggravates the patient condition 165, 166. The side effects of chemotherapy include digestive system reactions such as diarrhea and vomiting, myelosuppression, immune impairment, and liver and kidney damage. The most common reactions are digestive system reactions, which strongly implies that chemotherapy affects the gastrointestinal flora. Panebianco et al. evaluated whether gemcitabine treatment can affect the intestinal bacterial composition of mice that were xenografted with pancreatic cancer 167. The results showed that the proportion of gram-positive *Firmicutes* (from approximately 39 to 17%) and gram-negative *Bacteroidetes* (from 38 to 17%) in mouse intestines in the tumor-bearing control group decreased significantly, while the proportions of *Proteobacteria* (*E. coli* and *Aeromonas hydrophila*) and *Verrucomicrobia* (*Akkermansia muciniphila*) increased dramatically 167. Gram-positive *Firmicutes* and gram-negative *Bacteroidetes* are usually the dominant species in the gut of tumor-bearing control mice, while *Proteobacteria* and* Verrucomicrobia* are usually minor components 168-170. According to the analysis, the increase in *Proteobacteria* and *Verrucomicrobia* was related to inflammation of the intestine 171, 172. Researchers analyzed the serum metabolites of mice and found that those receiving gemcitabine had highly significant decreases in creatinine, which has anti-inflammatory and immunosuppressive properties 173, 174. In addition, the application of antibiotics to chemotherapy patients has also received attention from researchers. Previously, Geller et al. found that in a colorectal cancer model, when combined with gemcitabine, the antibiotic ciprofloxacin could eliminate the chemotherapy resistance caused by the bacteria in the tumor, significantly enhancing the treatment effect 150. This finding suggests that the combination of antibiotics and chemotherapy may be extremely beneficial in pancreatic cancer therapy. To explore the application of antibiotics during chemotherapy, some scientists retrospectively analyzed the indexes of 169 patients with advanced cancer (including pancreatic cancer) who received gemcitabine treatment. The patients were divided into two groups: an antibiotic-free group (treated with a gemcitabine-containing regimen but not treated with antibiotics) and an antibiotic treatment group (treated with a gemcitabine-containing regimen plus antibiotics). The effective rate, progression-free survival and overall survival of each group were evaluated. The results showed that the median progression-free survival and median overall survival of the antibiotic treatment group were longer than those of the antibiotic-free group, which indicates that adding antibiotics can improve the efficacy of chemotherapy in patients with advanced cancer 175. However, antibiotics also cause some side effects, such as adverse gastrointestinal events. Corty et al. used gemcitabine to treat 430 patients with metastatic pancreatic cancer. They used the Anderson-Gill survival model to compare the risk of adverse events between patients receiving antibiotics and those not receiving antibiotics. The results showed that receiving antibiotics was related to an increased risk of gemcitabine-associated, dose-limiting adverse events, including adverse gastrointestinal and hematological events 176. The survival period of patients who discontinued treatment due to adverse events was shorter than that of patients who continued treatment until the disease progressed. This finding indicates that antibiotics must be used cautiously in pancreatic cancer treatment and may actually be detrimental 176. Interestingly, the application of antibiotics in treating other cancers also supports this hypothesis. In mouse sarcoma, melanoma, and colon cancer models, a cocktail of the antibiotics ampicillin, colistin, and streptomycin renders cytotoxic lymphocyte antigen 4 (CTLA-4) treatment ineffective 177. Similarly, Viaud et al. found that pretreating mice receiving cyclophosphamide (CTX) with the gram-positive bacteria-targeting antibiotic vancomycin failed to activate the antitumor immune response, resulting in treatment failure 178. To conclude, chemotherapy can influence intestinal flora homeostasis. In addition, chemotherapy combined with antibiotics has immeasurable potential of showing its effectiveness against pancreatic cancer, but monitoring for possible side effects and adverse effects regarding the effectiveness of chemotherapy drugs during administration is indispensable.

## Prospects for use of microorganisms in pancreatic cancer chemotherapy

Research on the relationship between microbes and tumors has made noticeable progress, which will surely bring new promise for the treatment of pancreatic cancer. Oncolytic viruses, which are microorganisms that can be manipulated by humans, have unpredictable clinical prospects in pancreatic cancer treatment. As we mentioned above, many oncolytic viruses have significant synergistic effects when used in combination with chemotherapy to treat pancreatic cancer. However, there are still many directions worth exploring. For example, how can genetic modification achieve greater tumor selectivity and immunogenicity? How can we minimize the toxicity and maximize the activity of oncolytic viruses? How can we reduce virus loss before reaching the target site? When we combine chemotherapy and oncolytic viruses, which order of administration and dosage have the best effect? In the future, oncolytic viruses that interfere with the tumor microenvironment may reduce the number of resistant cases and the number of cancer stem cells. At present, most oncolytic viruses are delivered by intratumoral injection, and some experiments are guided through endoscopic ultrasound (EUS) 104. Determining which is the most appropriate delivery method is also warranted. Many clinical trials of oncolytic virus therapy are ongoing (representative clinical trials of oncolytic viruses with gemcitabine as a treatment for pancreatic cancer are listed in Table 1), and perhaps in the near future, oncolytic virus therapy will become the most promising treatment for pancreatic cancer. Our previous discussions show that bacteria in the tumor and gastrointestinal tract may have a vital impact on the effect of chemotherapy, so targeting relevant bacteria as a therapeutic strategy may induce different effects. The use of antibiotics in combination with chemotherapy has advantages and disadvantages. How to optimize these combinations to bring about better results warrants further exploration. Similarly, the use of chemotherapy to treat pancreatic cancer may induce a series of adverse effects by affecting the intestinal bacteria. An improved understanding of these concepts may lead to a better prognosis for patients with pancreatic cancer. Pharmacomicrobiomics, which can be used to evaluate the interaction between drugs and microorganisms, has become a popular topic in research and the clinic 152, 179. The use of bacteria as biomarkers for treating pancreatic cancer with chemotherapy is a potential strategy that can be studied in the future. In this way, we can monitor changes in treatment effects and the occurrence of chemotherapy resistance by monitoring changes in bacteria, which can ultimately be used to guide dose adjustments in parallel.

## Conclusions

Pancreatic cancer is a malignant neoplasm with poor prognosis, and gemcitabine is the primary chemotherapy drug for advanced pancreatic carcinoma. Currently, plenty of evidence suggests that there is an intricate interplay between microorganisms and pancreatic cancer. However, attention is primarily focused on how microorganisms lead to the occurrence and development of pancreatic cancer, and few studies have focused on elaborating on and summarizing the impact of microorganisms on pancreatic cancer chemotherapy. In this review, we provide new insights into the interplay of microorganisms and chemotherapy, summarizing some meaningful views and guidance for the development of new treatment methods and patterns. Our results indicate that the combination of oncolytic viruses and chemotherapy has great potential utility, and its mechanism possesses four characteristics: apoptosis of tumor cells, activation of antitumor immunity, increased sensitivity of cancer cells to chemotherapy and direct oncolysis. In addition, the use of bacteria in the treatment of pancreatic cancer has beneficial and negative effects on patient prognosis; chemotherapy may also interact with bacteria and cause various results, and antibiotics might have therapeutic potential in pancreatic cancer treatment. Surprisingly, fungi can also contribute to the occurrence of pancreatic cancer, but their influence and application during chemotherapy still require investigation. In addition, it should be noted that most of the studies in the above discussion were conducted in mouse models. Because there will likely be different effects during applications in humans, the results should be interpreted with caution. Overall, microorganisms are of paramount importance to pancreatic cancer chemotherapy, although the complex relationship between microorganisms, chemotherapy and pancreatic cancer remains unclear. In the near future, microorganisms will surely become highly important in pancreatic cancer treatment and new drug development.

## Figures and Tables

**Table 1:** Representative clinical trials of oncolytic viruses with gemcitabine as treatment for pancreatic cancer

Virus category	Virus names	Study phase	Structural modification	Chemotherapy intervention	Outcome measures	Clinical Trial ID/reference
oncolytic adenovirus	LOAd703	I/II	EIACR2 deletion, CD40L and 4-1BBL insertion	yes	Overall Response Rate Overall Survival	NCT02705196
oncolytic adenovirus	VCN-01	I	EIACR2 deletion, PH20 hyaluronidase addition	yes	Recommended Phase 2 Dose (RP2D) of VCN-01	NCT02045589
oncolytic adenovirus	VCN-01	I	EIACR2 deletion, PH20 hyaluronidase addition	yes	Safety and Tolerability, Presence of VCN-01 in tumor	NCT02045602
oncolytic adenovirus	ONYX-015	I/II	E1B55K gene deletion	yes	treatment effect	65
herpesviruses	OrienX010	I	Recombinant* hGM-CSF*	no	preliminary efficacy	NCT01935453
herpesviruses	HF10	I	*UL56* deletion	yes	Dose limiting toxicity (DLT) Adverse events (AEs)	NCT03252808
herpesviruses	HF10	I	*UL56* deletion	yes	safety assessment	104
herpesviruses	T-VEC	I	*ICP34.5* and *ICP47* Deletions, *GM-CSF* Insertion	no	Change in size of injected lesion(s),Overall response rate	NCT03086642
herpesviruses	T-VEC	I	*ICP34.5* and *ICP47* Deletions, *GM-CSF* Insertion	no	Adverse Events, (HSV-1) Antibodies	NCT00402025
Vaccinia virus	PANVAC-F plus PANVAC-V	I	None	no	MTD of falimarev. T cell proliferation, Cytokine production	NCT00669734
Vaccinia virus	MVAp53	I	Express WT murine *p53*	no	Safety and tolerance, Immunogenicity	NCT01191684
Vaccinia virus	p53MVA	I	Express *p53* and pembrolizumab	no	Tolerability,	NCT02432963
reoviruses	Reolysin	II	None	yes	clinical benefit rate, safety and tolerability	NCT00998322

## Abbreviations

HSVs: Herpes simplex viruses
FDA: Food and Drug Administration
CDD: Cytidine deaminase
E. coli: Escherichia coli
F. nucleatum: *Fusobacterium nucleatum*
TLR4: Toll-like receptor 4
MYD88: myeloid differentiation factor 88
CTX: cyclophosphamide
CTLA4: cytotoxic lymphocyte antigen 4
Ad5: adenovirus serotype 5
RGD: Arg-Gly-Asp
TMZ-CD40L: trimerized, membrane-bound human CD40 ligand
4-1BBL: 4-1BB ligand
ICP-6: infected cell polypeptide 6
PSCA: prostate stem cell antigen
GFP: green fluorescent protein
ORF: open reading frame
EUS: endoscopic ultrasound
DDR: DNA damage response
ECM: extracellular matrix
IL-10: interleukin-10
TGFβ1: transforming growth factor β1
Tregs: regulatory T cells
MDSCs: myeloid-derived suppressor cells
DAMPs: damage-associated molecular patterns
HMGB1: high mobility group box 1
PAMPs: pathogen-associated molecular patterns
TAAs: tumor-associated antigens
APCs: antigen-presenting cells
DCs: dendritic cells
and TME: tumor immune microenvironment
